# On the complexity of computing Markov perfect equilibrium in general-sum stochastic games

**DOI:** 10.1093/nsr/nwac256

**Published:** 2022-11-22

**Authors:** Xiaotie Deng, Ningyuan Li, David Mguni, Jun Wang, Yaodong Yang

**Affiliations:** Center on Frontiers of Computing Studies, School of Computer Science, Peking University, Beijing 100091, China; Center for Multi-Agent Research, Institute for AI, Peking University, Beijing 100091, China; Center on Frontiers of Computing Studies, School of Computer Science, Peking University, Beijing 100091, China; Huawei UK, London WC1E 6BT, UK; Computer Science, University College London, London WC1E 6BT, UK; Center for Multi-Agent Research, Institute for AI, Peking University, Beijing 100091, China

**Keywords:** Markov game, multi-agent reinforcement learning, Markov perfect equilibrium, **PPAD**-completeness, stochastic game

## Abstract

Similar to the role of Markov decision processes in reinforcement learning, Markov games (also called stochastic games) lay down the foundation for the study of multi-agent reinforcement learning and sequential agent interactions. We introduce approximate Markov perfect equilibrium as a solution to the computational problem of finite-state stochastic games repeated in the infinite horizon and prove its **PPAD**-completeness. This solution concept preserves the Markov perfect property and opens up the possibility for the success of multi-agent reinforcement learning algorithms on static two-player games to be extended to multi-agent dynamic games, expanding the reign of the **PPAD**-complete class.

## Teaser

We proved that solving Markov perfect equilibria of general-sum stochastic games is PPAD-complete, which lays down the foundation of algorithmic complexity for multi-agent reinforcement learning methodology.

## INTRODUCTION

Shapley [1] introduced stochastic games (SGs) to study the dynamic non-cooperative multi-player game, where each player simultaneously and independently chooses an action at each round for a reward. According to their current state and the chosen actions, the next state is determined by a probability distribution specified *a priori*. Shapley’s work includes the first proof of the existence of a stationary strategy profile under which no agent has an incentive to deviate, in two-player zero-sum SGs. Next, the existence of equilibrium in stationary strategies was extended to multi-player general-sum SGs by Fink [2]. Such a solution concept (known as *Markov perfect equilibrium* (MPE) [3]) captures the dynamics of multi-player games.

Because of its generality, the framework of SGs has enlightened a sequence of studies [4] on a wide range of real-world applications ranging from advertising and pricing [5], species interaction game modeling in fisheries [6], traveling inspection [7] and gaming AIs [8]. As a result, developing algorithms to compute MPE in SGs has become one of the key subjects in this extremely rich research domain, using approaches from applied mathematics, economics, operations research, computer science and artificial intelligence (see, e.g., [9]).

The concept of the SG underpins many AI and machine learning studies. The optimal policy making of Markov decision processes (MDPs) captures the central problem of a single agent interacting with its environment, according to Sutton and Barto [10]. In multi-agent reinforcement learning (MARL) [11,12], SG extends to incorporate the dynamic nature in multi-agent strategic interactions, to study optimal decision making and subsequently equilibria in multi-player games [13,14].

For two-player zero-sum (discounted) SGs, the game-theoretical equilibrium is closely related to the optimization problem in MDPs as the opponent is purely adversarial [15,16]. On the other hand, solving general-sum SGs has been possible only under strong assumptions [2,17]. Zinkevich *et al.* [18] demonstrated that, for the entire class of value iteration methods, it is difficult to find stationary Nash equilibrium (NE) policies in general-sum SGs. This has led to few existing MARL algorithms to general-sum SGs. Known approaches have either studied special cases of SGs [19,20] or ignored the dynamic nature to limit the study to the weaker notion of Nash equilibrium [21].

Recently, Solan and Vieille [22] reconfirmed the importance of the existence of a stationary strategy profile as having several philosophical implications. First, it is conceptually straightforward. Second, *past play affects the players’ future behavior only through the current state*. Third, subsequently and most importantly, *equilibrium behavior does not involve non-credible threats, a property that is stronger than the equilibrium property, and viewed as highly desirable* [23].

Surprisingly, the complexity of finding an MPE in an SG remains an open problem, although an SG was proposed more than sixty years ago and despite its importance. While fruitful studies have been conducted on zero-sum SGs, we still know little about the complexity of solving general-sum SGs. It is clear that solving MPE in (infinite-horizon) SGs is at least **PPAD**-hard, since solving a two-player NE in one-shot SGs is already complete in this computational class [24,25] defined by Papadimitriou [26]. This suggests that it is unlikely to have polynomial-time algorithms in general-sum stochastic games for two players. Yet, with complications involved in the general-sum and dynamic settings, the unresolved challenge has been: Can solving MPE in general-sum SGs be anywhere complete in computational complexity classes?

We answer the above question in the positive, proving the computation of an approximate MPE in SGs equivalent to that of a Nash equilibrium in a single state setting, and subsequently showing its **PPAD**-completeness. It opens up the possibility to develop MARL algorithms to work for the general-sum SGs in the same way as for an ordinary Nash equilibrium computation.

### Intuitions and a sketch of our main ideas

Computational studies on problem solving build understanding on various types of reduction. After all, computations carried out on computers are eventually reduced to AND/OR/NOT gates on electronic circuits.

To prove that a problem is **PPAD**-complete, we need to prove that it is in the class, and that it can be used as a base to solve any other problem in this class (for its hardness). More formally, the reduction needs to be carried out in polynomial (with respect to the input size) time. Nash equilibrium computation of two-player normal-form games [27] is arguably the most prominent **PPAD**-complete problem [24,25]. When one stochastic game has only one state and the discount factor γ = 0, then finding an MPE is equivalent to finding a Nash equilibrium in the corresponding normal-form game. The **PPAD**-hardness of finding an MPE follows immediately. Our main result is to prove the **PPAD** membership property of computing an approximate MPE (Lemma 2 below).

Firstly, we construct a function *f* on the strategy profile space, such that each strategy profile is a fixed point of *f* if and only if it is an MPE of the stochastic game (Theorem 2 below). Furthermore, we prove that the function *f* is continuous (λ-Lipschitz by Lemma 3 below), so that fixed points are guaranteed to exist by the Brouwer fixed point theorem.

Secondly, we prove that the function *f* has some ‘good’ approximation properties. Let $|\mathcal {SG}|$ be the input size of a stochastic game. If we can find a $\mathrm{poly}(|\mathcal {SG}|)\epsilon ^2$-approximate fixed point π of *f*, i.e. $\Vert f(\pi )-\pi \Vert _{\infty }\le \mathrm{poly}(|\mathcal {SG}|)\epsilon ^2$, where π is a strategy profile, then π is an ε-approximate MPE for the stochastic game (combining Lemma 5 and Lemma 6 below). So our goal converts to finding an approximate fixed point of a Lipschitz function.

Finally, our **PPAD** membership follows from the theorem that computation of the approximate Brouwer fixed point of a Lipschitz function is **PPAD**-complete, as shown in the seminal paper by Papadimitriou [26].

### Related work

In practice, MARL methods are most often applied to compute the MPE of an SG based on the interactions between agents and the environment. Their uses can be classified in two different settings: *online* and *offline*. In the offline setting (also known as the batch setting [21]), the learning algorithm controls all players in a centralized way, hoping that the learning dynamics can eventually lead to an MPE by using a limited number of interaction samples. In the online setting, the learner controls only one of the players to play with an arbitrary group of opponents in the game, assuming unlimited access to the game environment. The central focus is often on the *regret*: the difference between the learner’s total reward during learning versus that of a benchmark measure in hindsight.

In the offline setting, two-player zero-sum (discounted) SGs have been extensively studied. Since the opponent is purely adversarial in zero-sum SGs, the process of seeking the worst-case optimality for each player can be thought of as solving MDPs. As a result, (approximate) dynamic programming methods [28,29] such as least-squares policy iteration [30] and fitted value iteration [31] or neural fitted Q iteration [32] can be adopted to solve SGs [33–36]. Under this setting, policy-based methods [37,38] can also be applied. However, directly applying existing MDP solvers on general-sum SGs is problematic. Since solving two-player NE in general-sum normal-form games (i.e. one-shot SGs) is well known to be **PPAD**-complete [24,25], the complexity of MPE in general-sum SGs is expected to be at least **PPAD**-hard. Although early attempts such as Nash-Q learning [39], correlated-Q learning [40], friend-or-foe Q-learning [41] have been made to solve general-sum SGs under strong assumptions, Zinkevich *et al.* [18] demonstrated that none in the entire class of value iteration methods can find stationary NE policies in general-sum SGs. The difficulties on both the complexity side and the algorithmic side have led to few existing MARL algorithms for general-sum SGs. Successful approaches either assume knowing the complete information of the SG such that solving MPE can be turned into an optimization problem [42], or prove the convergence of batch RL methods to a weaker notion of NE [21].

In the online setting, agents aim to minimize their regret by trial and error. One of the most well-known online algorithms is R-MAX [43], which studies (average-reward) zero-sum SGs and provides a polynomial (in game size and error parameter) regret bound while competing against an arbitrary opponent. Following the same regret definition, UCSG [44] improved R-MAX and achieved a sublinear regret, but still in the two-player zero-sum SG setting. When it comes to MARL solutions, Littman [13] proposed a practical solution named Minimax-Q that replaces the max operator with the minimax operator. Asymptotic convergence results of Minimax-Q were developed in both tabular cases [45] and value function approximations [46]. To avoid the overly pessimism property by playing the minimax value for general-sum SGs, WoLF [47] was proposed to take variable steps to exploit an opponent’s suboptimal policy for a higher reward on a variety of stochastic games. AWESOME [48] further generalized WoLF and achieved NE convergence in multi-player general-sum repeated games. However, outside the scope of zero-sum SGs, the question [43] of whether a polynomial time no-regret (near-optimal) MARL algorithm exists for general-sum SGs remains open.

Some recent works studied the sample complexity issue in RL and MARL algorithms, most of which considered a finite horizon. Jin *et al.* [49] proved that a variant of Q-learning with upper confidence bound exploration can achieve a near-optimal sample efficiency under episodic MDP setting. Zhang *et al.* [50] proposed a learning algorithm for episodic MDP with a regret bound close to the information theoretic lower bound. Li *et al.* [51] proposed a probably approximately correct learning algorithm for episodic RL with a sample complexity independent of the planning horizon. For general-sum MARL, Chen *et al.* [52] proved an exponential lower bound on the sample complexity of approximate Nash equilibrium even in *n*-player normal-form games. In the same direction, Song *et al.* [53] showed that correlated equilibrium (CE) and coarse correlated equilibrium (CCE) can be learned within a sample complexity polynomial in the maximum size of the action set of a player, rather than the size of the joint action space. Jin *et al.* [54] developed a decentralized MARL algorithm with polynomial sample complexity to learn CE and CCE.

## DEFINITIONS AND THE MAIN THEOREM

**Definition 1** (Stochastic game). A stochastic game is defined by a tuple of six elements $\langle n,\mathbb {S},\mathbb {A},P,r,\gamma \rangle$.

- By *n* we denote the number of agents.
- By $\mathbb {S}$ we denote the set of finite environmental states. Let $S=|\mathbb {S}|$.
- By $\mathbb {A}^i$ we denote the action space of agent *i*. Note that each agent *i* can choose different actions under different states. Without loss of generality, we assume that, for each agent *i*, the action space $\mathbb {A}^i$ under each state is the same. Here $\mathbb {A}=\mathbb {A}^1\times \cdots \times \mathbb {A}^n$ is the set of agents’ joint action vector. Let $A^i=|\mathbb {A}^i|$ and *A*_max _ = max _*i* ∈ [*n*]_*A*^*i*^.
- By $P: \mathbb {S}\times \mathbb {A}\rightarrow \Delta (\mathbb {S})$ we denote the transition probability, that is, at each time step, given the agents’ joint action vector $a\in \mathbb {A}$, then the transition probability from state *s* to state *s*′ in the next time step is *P*(*s*′|*s, a*).
- By $r=r^1\times \cdots \times r^n: \mathbb {S}\times \mathbb {A}\rightarrow \mathcal {R}_+^n$ we denote the reward function, that is, when agents are at state *s* and play the joint action vector *a*, then agent *i* will get reward *r*^*i*^(*s, a*). We assume that the rewards are bounded by *r*_max _.
- By γ ∈ [0, 1) we denote the discount factor that specifies the degree to which the agent’s rewards are discounted over time.

Each agent aims to find a behavioral strategy with Markovian property, which is conditioned on the current state of the game.

The pure strategy space of agent *i* is $\prod _{s\in \mathbb {S}}\mathbb {A}^i$, which means that agent *i* needs to select an action at each state. Note that the size of the pure strategy space of each agent is $|\mathbb {A}^i|^S$, which is already exponential in the number of states. More generally, we define the mixed behavioral strategy as follows.

**Definition 2** (Behavioral strategy). A behavioral strategy of agent *i* is $\pi ^i: \mathbb {S}\rightarrow \Delta (\mathbb {A}^i)$. For all $s\in \mathbb {S}$, π^*i*^(*s*) is a probability distribution on $\mathbb {A}^i$.

In the rest of the paper, we focus on behavioral strategy and refer to it simply as a strategy for convenience. A strategy profile π is the Cartesian product of all agents’ strategies, i.e. π = π^1^ × ⋅⋅⋅ × π^*n*^. We denote the probability of agent *i* using action *a*^*i*^ at state *s* by π^*i*^(*s, a*^*i*^). The strategy profile of all the agents other than agent *i* is denoted by π^−*i*^. We use π^*i*^, π^−*i*^ to represent π, and *a*^*i*^, *a*^−*i*^ to represent *a*.

Given π, the transition probability and the reward function depend only on the current state $s\in \mathbb {S}$. Let *r*^*i*, π^(*s*) denote $\mathbb {E}_{a\sim \pi (s)}[r^i(s,a)]$ and *P*^π^(*s*′|*s*) denote $\mathbb {E}_{a\sim \pi (s)}[P(s^{\prime }|s,a)]$. Fix π^−*i*^; the transition probability and the reward function depend only on the current state $s\in \mathbb {S}$ and player *i*’s action *a*^*i*^. Let $r^{i,\pi ^{-i}}(s,a^i)$ denote $\mathbb {E}_{a^{-i}\sim \pi ^{-i}(s)}[r^i(s,(a^i,a^{-i}))]$ and $P^{\pi ^{-i}}(s^{\prime }|s,a^i)$ denote $\mathbb {E}_{a^{-i}\sim \pi ^{-i}(s)}[P(s^{\prime }|s,(a^i,a^{-i}))]$.

For any positive integer *m*, let $\Delta _m:=\lbrace x\in \mathcal {R}_+^m|\sum _{i=1}^m x_i=1\rbrace$. Define $\Delta _{A^i}^S:=\Pi _{s\in \mathbb {S}}\Delta _{A^i}$. Then, for all $s\in \mathbb {S},\pi ^i(s)\in \Delta _{A^i}$, $\pi ^i\in \Delta _{A^i}^S$ and $\pi \in \prod _{i=1}^n\Delta _{A^i}^S$.

**Definition 3** (Value function). A value function for agent *i* under strategy profile π, $V_i^{\pi }(s):\mathbb {S}\rightarrow R$, gives the expected sum of its discounted rewards when the starting state is *s*:

$$\begin{eqnarray*}
V_i^{\pi }(s)= \sum _{t=0}^{\infty } \gamma ^t\cdot \mathop {\mathbb {E}}[r^{i,\pi }(s_{t})|s_0=s].
\end{eqnarray*}$$

Here, *s*_0_, *s*_1_, … is the Markov chain such that the transition matrix is *P*^π^, that is, $\Pr [s_{k+1}=s^{\prime }|s_k=s]=P^\pi (s^{\prime }|s)$ for all *k* = 0, 1, …. Equivalently, the value function can be defined recursively via the Bellman policy equation 

$$\begin{eqnarray*}
&&V_i^{\pi }(s)\nonumber\\
&&= \mathop {\mathbb {E}}_{a\sim \pi (s)}\bigg [ r^{i}(s,a)+\gamma \sum _{s^{\prime }\in \mathbb {S}} P(s^{\prime }|s,a)V_i^{\pi }(s^{\prime })\bigg ].
\end{eqnarray*}$$

**Definition 4** (Markov perfect equilibrium). A behavioral strategy profile π is called a Markov perfect equilibrium if, for all $s\in \mathbb {S}$, all *i* ∈ [*n*] and all $\tilde{\pi }^i\in \Delta _{A^i}^S$,

$$\begin{equation*}
V_i^{\pi }(s)\ge V_i^{\tilde{\pi }^i,\pi ^{-i}}(s),
\end{equation*}$$

where $V_i^{\tilde{\pi }^i,\pi ^{-i}}(s)$ is the value function of *i* when its strategy deviates to $\tilde{\pi }^i$ while the strategy profile of other agents is π^−*i*^.

The Markov perfect equilibrium is a solution concept within SGs in which the players’ strategies depend only on the current state but not on the game history.

**Definition 5** (ε-approximate MPE). Given ε > 0, a behavioral strategy profile π is called an ε-approximate MPE if, for all $s\in \mathbb {S}$, all *i* ∈ [*n*] and all $\tilde{\pi }^i\in \Delta _{A^i}^S$,

$$\begin{equation*}
V_i^{\pi }(s)\ge V_i^{\tilde{\pi }^i,\pi ^{-i}}(s)-\epsilon .
\end{equation*}$$

We use Approximate MPE to denote the computational problem of finding an approximate Markov perfect equilibrium in stochastic games, where the inputs and outputs are as follows. The input instance of problem Approximate MPE is a pair $(\mathcal {SG},L)$, where $\mathcal {SG}$ is a stochastic game and *L* is a positive integer. The output of problem Approximate MPE is a strategy profile $\pi \in \prod _{i=1}^n\Delta _{A^i}^S$, also dependent only at the current state but not on its history, such that π is a 1/*L*-approximate MPE of $\mathcal {SG}$. We use the notation $|\mathcal {SG}|$ to denote the input size of the stochastic game $\mathcal {SG}$.

**Theorem 1** (Main theorem). Approximate MPE*is PPAD-complete*.

We note that, when |*S*| = 1 and γ = 0, a stochastic game degenerates to an *n*-player normal-form game. At this time, any MPE of this stochastic game is a Nash equilibrium for the corresponding normal-form game. So we have the following hardness result immediately.

**Lemma 1.** Approximate MPE *is PPAD-hard*.

To derive Theorem 1, we focus on the proof of **PPAD** membership of Approximate MPE in the rest of the paper.

**Lemma 2.** Approximate MPE *is in PPAD*.

## ON THE EXISTENCE OF MPE

The original proof of the existence of MPE is from [2] and based on Kakutani’s fixed point theorem. Unfortunately, proofs that are based on Kakutani’s fixed point theorem in general cannot be turned into **PPAD**-membership results. We develop a proof that uses Brouwer’s fixed point theorem, based on which we also prove the **PPAD** membership of Approximate MPE.

Inspired by the continuous transformation defined by Nash to prove the existence of the equilibrium point [27], we define an updating function $f: \prod _{i=1}^n\Delta _{A^i}^S\rightarrow \prod _{i=1}^n\Delta _{A^i}^S$ to adjust the strategy profile of agents in a stochastic game to establish the existence of MPE.

Let $\pi \in \prod _{i=1}^n\Delta _{A^i}^S$ be the behavioral strategy profile under discussion.

Let $Q_i^{\pi ^i,\pi ^{-i}}(s,a^i)$ denote the expected sum of discounted rewards of agent *i* if agent *i* uses pure action *a*^*i*^ at state *s* at the first step, and then follows π^*i*^ after that, but every other agent *j* maintains its strategy π^*j*^. Formally,

$$\begin{eqnarray*}
&&\!\!\!\!Q_i^{\pi ^i,\pi ^{-i}}(s,a^i)\\ &&\!\!\!\!=r^{i,\pi ^{-i}}(s,a^i)
+\,\gamma\! \sum _{s^{\prime }\in \mathbb {S}}\!P^{\pi ^{-i}}(s^{\prime }|s,a^i)V_i^{\pi ^i,\pi ^{-i}}(s^{\prime }).
\end{eqnarray*}$$

For each player *i* ∈ [*n*] with each action $a^i\in \mathbb {A}^i$ at each state $s\in \mathbb {S}$, we define a policy update function of π^*i*^(*s, a*^*i*^) as

$$\begin{eqnarray*}
&&f(\pi )^i(s,a^i)\nonumber\\
&&=\frac{\pi ^i(s,a^i)\!+\!\max \left(0,Q_i^{\pi ^i,\pi ^{-i}}(s,a^i)\!-\!V_i^{\pi ^i,\pi ^{-i}}(s) \right)}{1\!+\!\sum _{b^i\in \mathbb {A}^i}\max \left(0,Q_i^{\pi ^i,\pi ^{-i}}(s,b^i)\!-\!V_i^{\pi ^i,\pi ^{-i}}(s)\right)}.
\end{eqnarray*}$$

We consider the infinite norm distance of two strategy profiles π_1_ and π_2_, denoted by ‖π_1_ − π_2_‖_∞_: $\Vert \pi _1-\pi _2\Vert _{\infty }=\max _{i\in [n], s\in \mathbb {S},a^i\in \mathbb {A}^i}|\pi _1^i(s,a^i)-\pi _2^i(s,a^i)|$.

We first prove that the function *f* satisfies a continuity property, namely, is *λ-Lipschitz* for λ equal to ${11nS^2A_{\max }^2r_{\max }}/{(1-\gamma )^2}$.

**Lemma 3.** *The function f is* λ-*Lipschitz, i.e. for every*$\pi _1,\pi _2\in \prod _{i=1}^n\Delta _{A^i}^S$*such that* ‖π_1_ − π_2_‖_∞_ ≤ δ, *we have*

$$\begin{equation*}
\!\!\!\!\Vert f(\pi _1)-f(\pi _2)\Vert _{\infty }\le \frac{11nS^2A_{\max }^2r_{\max }}{(1-\gamma )^2} \delta .
\end{equation*}$$

*Proof.* At any $s\in \mathbb {S}$, pick any player *i* ∈ [*n*]. For an action $a^i\in \mathbb {A}^i$, let *M*_1_(*a*^*i*^) denote $\max (0,Q_i^{\pi _1^i, \pi _1^{-i}}(s,a^i)-V_i^{\pi _1^i,\pi _1^{-i}}(s))$ and $M_2(a^i)= \max (0,Q_i^{\pi _2^i,\pi _2^{-i}}(s,a^i)-V_i^{\pi _2^i,\pi _2^{-i}}(s))$. From the next claim (proof in the Appendix),

$$\begin{eqnarray*}
&& |f(\pi _1)^i(s,a^i)-f(\pi _2)^i(s,a^i)| \\
&& = \bigg |\frac{\pi _1^i(s,a^i)+M_1(a^i)}{1+\sum _{b^i\in \mathbb {A}^i}M_1(b^i)} - \frac{\pi _2^i(s,a^i)+M_2(a^i)}{1+\sum _{b^i\in \mathbb {A}^i}M_2(b^i)}\bigg | \\
&&\le |\pi _1^i(s,a^i)-\pi _2^i(s,a^i)| + |M_1(a^i)-M_2(a^i)| \\
&& +\, \bigg |\sum _{b^i\in \mathbb {A}^i}M_1(b^i)- \sum _{b^i\in \mathbb {A}^i}M_2(b^i)\bigg |.
\end{eqnarray*}$$

**Claim 1.** *For any x, x*′, *y, y*′, *z, z*′ ≥ 0 *such that* (*x* + *y*)/(1 + *z*) ≤ 1 *and* (*x*′ + *y*′)/(1 + *z*′) ≤ 1, *it holds that*

$$\begin{eqnarray*}
&&\bigg |\frac{x+y}{1+z}-\frac{x^{\prime }+y^{\prime }}{1+z^{\prime }}\bigg |\le |x-x^{\prime }| +\, |y-y^{\prime }|\\
&&\qquad+\,|z-z^{\prime }|.
\end{eqnarray*}$$

Take δ = ‖π_1_ − π_2_‖_∞_; then $|\pi _1^i(s,a^i)-\pi _2^i(s,a^i)|\le \delta$ for any $s\in \mathbb {S}, a^i\in \mathbb {A}^i$. Next, for any $a^i\in \mathbb {A}^i$, we estimate

$$\begin{eqnarray*}
&&\vert M_1(a^i)-M_2(a^i)\vert \\
&&\quad =\left\vert\, \max \left(0,Q_i^{\pi _1^i,\pi _1^{-i}}(s,a^i)-V_i^{\pi _1^i,\pi _1^{-i}}(s)\right)\right.\\
&&- \left.\max \left(0,Q_i^{\pi _2^i,\pi _2^{-i}}(s,a^i)-V_i^{\pi _2^i,\pi _2^{-i}}(s)\right)\right\vert \\
&&\quad \le \left|\left(Q_i^{\pi _1^i,\pi _1^{-i}}(s,a^i)-V_i^{\pi _1^i,\pi _1^{-i}}(s)\right)\right.\\
&&- \left.\left(Q_i^{\pi _2^i,\pi _2^{-i}}(s,a^i)-V_i^{\pi _2^i,\pi _2^{-i}}(s)\right)\right|\\
&&\quad \le \left|Q_i^{\pi _1^i,\pi _1^{-i}}(s,a^i)-Q_i^{\pi _2^i,\pi _2^{-i}}(s,a^i)\right|\\
&&\quad \quad + \left|V_i^{\pi _1^i,\pi _1^{-i}}(s)-V_i^{\pi _2^i,\pi _2^{-i}}(s)\right|.
\end{eqnarray*}$$

We should first derive an upper bound on $|r^{i,\pi _1^{-i}}(s,b^i)-r^{i,\pi _2^{-i}}(s,b^i)|$.

**Claim 2.** *It holds that*

$$\begin{eqnarray*}
\left|r^{i,\pi _1^{-i}}(s,b^i)\!-\!r^{i,\pi _2^{-i}}(s,b^i)\right|\! \le\! (n\!-\!1)A_{\max }r_{\max }\delta .
\end{eqnarray*}$$

This follows from the following claim (proof in the Appendix).

**Claim 3.** *It holds that*

$$\begin{eqnarray*}
&&\sum _{b^{-1}\in \mathbb {A}^{-1}} \bigg |\prod _{j=2}^n\pi _1^{j}(s,b^j)-\prod _{j=2}^n\pi _2^{j}(s,b^j)\bigg |\\
&&\qquad \le (n-1)A_{\max }\delta .
\end{eqnarray*}$$

Similarly, we have the following claim.

**Claim 4.** *It holds that*

$$\begin{eqnarray*}
&&\sum _{b^{-1}\in \mathbb {A}^{-1}} |P^{\pi _1^{-i}}(s^{\prime }|s,b^i)-P^{\pi _2^{-i}}(s^{\prime }|s,b^i)|\\
&&\qquad \le (n-1)A_{\max }\delta .
\end{eqnarray*}$$

To bound $|V_i^{\pi ^i_1,\pi ^{-i}_1}(s)-V_i^{\pi ^i_2,\pi ^{-i}_2}(s)|$ for every $s\in \mathbb {S}$, we denote by $V_i^{\pi }$ the column vector $(V_i^{\pi }(s))_{s\in \mathbb {S}}$, and by *r*^*i*, π^ the column vector $(r^{i,\pi }(s))_{s\in \mathbb {S}}$ and by *P*^π^ the matrix $P^{\pi }(s,s^{\prime })_{s,s^{\prime }\in \mathbb {S}}$. By the Bellman policy equation (Definition 3), we have

$$\begin{equation*}
V_i^{\pi }=r^{i,\pi }+\gamma P^{\pi }V_i^{\pi },
\end{equation*}$$

which means that

$$\begin{equation*}
V_i^{\pi }=(I-\gamma P^{\pi })^{-1}r^{i,\pi }.
\end{equation*}$$

We prove in Lemma 7 below that

$$\begin{eqnarray*}
&&|(I-\gamma P^{\pi _1})^{-1}(s^{\prime }|s)-(I-\gamma P^{\pi _2})^{-1}(s^{\prime }|s)|\\
&&\qquad \le \frac{nSA_{\max }\delta }{(1-\gamma )^2}
\end{eqnarray*}$$

for all $s,s^{\prime }\in \mathbb {S}$.

Now we are ready to give an upper bound on $|V_i^{\pi ^i_1,\pi ^{-i}_1}(s)-V_i^{\pi ^i_2,\pi ^{-i}_2}(s)|$ for any $s\in \mathbb {S}$. We have

$$\begin{eqnarray*}
&&\left|V_i^{\pi ^i_1,\pi ^{-i}_1}(s)-V_i^{\pi ^i_2,\pi ^{-i}_2}(s)\right|\\
&&\quad = \bigg |\sum _{s^{\prime }\in \mathbb {S}}r^{i,\pi _1}(s^{\prime })(I-\gamma P^{\pi _1})^{-1}(s^{\prime }|s)\\
&&-\sum _{s^{\prime }\in \mathbb {S}}r^{i,\pi _2}(s^{\prime })(I-\gamma P^{\pi _2})^{-1}(s^{\prime }|s)\bigg |\\
&&\quad \le \sum _{s^{\prime }\in \mathbb {S}} r^{i,\pi _1}(s^{\prime }) |(I-\gamma P^{\pi _1})^{-1}(s^{\prime }|s)\\
&& -\, (I-\gamma P^{\pi _2})^{-1}(s^{\prime }|s)|\\
&&\quad + \sum _{s^{\prime }\in \mathbb {S}} (I\!-\!\gamma P^{\pi _2})^{-1}(s^{\prime }|s)\\
&& \times\left|r^{i,\pi _1}(s^{\prime })\!-\!r^{i,\pi _2}(s^{\prime })\right|\\
&&\quad \le \sum _{s^{\prime }\in \mathbb {S}}\bigg (r_{\max }\frac{nSA_{\max }\delta }{(1-\gamma )^2}+\frac{1}{1-\gamma } nA_{\max }r_{\max }\delta \bigg )\\
&&\quad = \frac{SnA_{\max }r_{\max }}{1-\gamma }\bigg (\frac{S}{1-\gamma }+1\bigg )\delta \\
&&\quad \le \frac{2nS^2A_{\max }r_{\max }}{(1-\gamma )^2}\delta ,
\end{eqnarray*}$$

where the forth line follows from $|(I-\gamma P^{\pi _2})^{-1} (s^{\prime }|s)|\le {1}/{(1-\gamma )}$, in Lemma 7 below.

Similarly, we establish a bound for $|Q_i^{\pi _1^i,\pi _1^{-i}}(s,b^i)-Q_i^{\pi _2^i,\pi _2^{-i}}(s,b^i)|$:

$$\begin{eqnarray*}
&& \left|Q_i^{\pi _1^i,\pi _1^{-i}}(s,b^i)-Q_i^{\pi _2^i,\pi _2^{-i}}(s,b^i) \right|\\
&& = \left |r^{i,\pi _1^{-i}}(s,b^i)
+\, \gamma \sum _{s^{\prime }\in \mathbb {S}}P^{\pi _1^{-i}}(s^{\prime }|s,b^i)V_i^{\pi _1^i,\pi _1^{-i}}(s^{\prime })\right.\\ &&- r^{i,\pi _2^{-i}}(s,b^i)
-\gamma \sum _{s^{\prime }\in \mathbb {S}}P^{\pi _2^{-i}}(s^{\prime }|s,b^i)V_i^{\pi _2^i,\pi _2^{-i}}(s^{\prime })\bigg | \\
&& \le |r^{i,\pi _1^{-i}}(s,b^i)-r^{i,\pi _2^{-i}}(s,b^i)|\\
&&\qquad +\,\gamma \sum _{s^{\prime }\in \mathbb {S}}\Big |P^{\pi _1^{-i}}(s^{\prime }|s,b^i)V_i^{\pi _1^i,\pi _1^{-i}}(s^{\prime })\\
&&\qquad-\,P^{\pi _2^{-i}}(s^{\prime }|s,b^i)V_i^{\pi _2^i,\pi _2^{-i}}(s^{\prime })\Big | \\
&& \le nA_{\max }r_{\max }\delta \\
&& +\, \gamma \sum _{s^{\prime }\in \mathbb {S}}\left(P^{\pi _1^{-i}}(s^{\prime }|s,b^i) \left|V_i^{\pi _1^i,\pi _1^{-i}}(s^{\prime })\right.\right.\\
&& -\,V_i^{\pi _2^i,\pi _2^{-i}}(s^{\prime })\left|+ V_i^{\pi _2^i,\pi _2^{-i}}(s^{\prime })\right|P^{\pi _1^{-i}}(s^{\prime }|s,b^i)\\
&&\left.\left.-\, P^{\pi _2^{-i}}(s^{\prime }|s,b^i)\right|\right) \\
&& \le nA_{\max }r_{\max }\delta \\
&& +\,\gamma \bigg (\frac{2nS^2A_{\max }r_{\max }}{(1-\gamma )^2}\delta + S\frac{r_{\max }}{1-\gamma }nA_{\max }\delta \bigg )\\
&& = nA_{\max }r_{\max }\delta \bigg (1+\frac{2\gamma S^2}{(1-\gamma )^2}+\frac{\gamma S}{1-\gamma }\bigg )\\
&& \le \frac{3S^2nA_{\max }r_{\max }\delta }{(1-\gamma )^2}.
\end{eqnarray*}$$

For any $b^i\in \mathbb {A}^i$, we have

$$\begin{eqnarray*}
&&\vert M_1(b^i)-M_2(b^i)\vert\\
&&\quad \le \left|Q_i^{\pi _1^i,\pi _1^{-i}}(s,b^i)-Q_i^{\pi _2^i,\pi _2^{-i}}(s,b^i)\right| \\
&&\qquad +\, \left|V_i^{\pi _1^i,\pi _1^{-i}}(s)-V_i^{\pi _2^i,\pi _2^{-i}}(s)\right| \\
&&\quad \le \frac{5S^2nA_{\max }r_{\max }\delta }{(1-\gamma )^2}.
\end{eqnarray*}$$

Thus, for any $s\in \mathbb {S}$ and any $a^i\in \mathbb {A}^i$, we obtain

$$\begin{eqnarray*}
&&|f(\pi _1)^i(s,a^i)-f(\pi _2)^i(s,a^i)|\\
&&\quad \le |\pi _1^i(s,a^i)-\pi _2^i(s,a^i)|\\
&&\qquad +\vert M_1(a^i) - M_2(a^i)\vert\\
&& +\sum _{b^i\in \mathbb {A}^i}\vert M_1(b^i)-M_2(b^i)\vert \\
&&\quad \!\!\!\le \delta +\frac{5S^2nA_{\max }r_{\max }\delta }{(1-\gamma )^2}+A_{\max }\frac{5S^2nA_{\max }r_{\max }\delta }{(1-\gamma )^2}\\
&&\quad \le \frac{11nS^2A_{\max }^2r_{\max }}{(1-\gamma )^2}\delta .
\end{eqnarray*}$$

This completes the proof of Lemma 3.□

Now we can establish the existence of MPE by the Brouwer fixed point theorem.

**Theorem 2.** *For any stochastic game* $\langle n,\mathbb {S},\mathbb {A}, P,r,\gamma \rangle$ , *a strategy profile* π *is an MPE if and only if it is a fixed point of the function f, i.e. f*(π) = π. *Furthermore, the function f has at least one fixed point*.

*Proof.* We first show that the function *f* has at least one fixed point. Brouwer’s fixed point theorem states that, for any continuous function mapping a compact convex set to itself, there is a fixed point. Note that *f* is a function mapping a compact convex set to itself. Also, *f* is continuous by Lemma 3. Therefore, the function *f* has at least one fixed point.

We then prove that a strategy profile π is an MPE if and only if it is a fixed point of *f*.

⇒: For the necessity, suppose that π is an MPE; then, by Definition 4, we have, for each player *i* ∈ [*n*], each state $s\in \mathbb {S}$ and each policy $\tilde{\pi }^i\in \Delta _{A^i}^S$, $V_i^{\pi ^i,\pi ^{-i}}(s)\ge V_i^{\tilde{\pi }^i,\pi ^{-i}}(s)$. By Lemma 4 to be proven next, we have, for any action $a^i\in \mathbb {A}^i$, $V_i^{\pi ^i,\pi ^{-i}}(s)\ge Q_i^{\pi ^i,\pi ^{-i}}(s,a^i)$, which implies that $\max (0,Q_i^{\pi ^i,\pi ^{-i}}(s,a^i)-V_i^{\pi ^i,\pi ^{-i}}(s))=0$. Then, for each player *i* ∈ [*n*], each state $s\in \mathbb {S}$ and each action $a^i\in \mathbb {A}^i$, (*f*(π))^*i*^(*s, a*^*i*^) = π^*i*^(*s, a*^*i*^). It follows that π is a fixed point of *f*.

⇐: For the proof of the sufficiency part, let π be a fixed point of *f*. Then, for each player *i* ∈ [*n*], each state $s\in \mathbb {S}$ and each action $a^i\in \mathbb {A}^i$,

$$\begin{eqnarray*}
&&\pi ^i(s,a^i)\\
&&=(f(\pi ))^i(s,a^i) \\
&&=\frac{\pi ^i(s,a^i)\!+\!\max \left(0,Q_i^{\pi ^i,\pi ^{-i}}(s,a^i)\!-\!V_i^{\pi ^i,\pi ^{-i}}(s)\right)}{1\!+\!\sum _{b^i\in \mathbb {A}^i}\max \left(0,Q_i^{\pi ^i,\pi ^{-i}}(s,b^i)\!-\!V_i^{\pi ^i,\pi ^{-i}}(s)\right)}.
\end{eqnarray*}$$

We first provide the following claim given the condition that π is a fixed point.

**Claim 5.** *For any* $a^i\in \mathbb {A}^i$ , $Q_i^{\pi ^i,\pi ^{-i}}(s,a^i)-V_i^{\pi ^i,\pi ^{-i}}(s)\le 0$.

*Proof of Claim 5.* Suppose for contradiction that there exists *i* ∈ [*n*] and $d^i\in \mathbb {A}^i$ such that $Q_i^{\pi ^i,\pi ^{-i}}(s,d^i)>V_i^{\pi ^i,\pi ^{-i}}(s)$. The above fixed point equation implies that π^*i*^(*s, d*^*i*^) > 0.

Let $\mathbb {A}^i_+=\lbrace a^i\in \mathbb {A}^i:\pi ^i(s,a^i)>0\rbrace$; then $d^i\in \mathbb {A}^i_+$. Note that, by the recursive definition of $V_i^{\pi ^i,\pi ^{-i}}(s)$, we have

$$\begin{eqnarray*}
V_i^{\pi ^i,\pi ^{-i}}(s) &=&\mathop {\mathbb {E}}_{a^i\sim \pi ^i(s)}\bigg [r^{i,\pi ^{-i}}(s,a^i)\\
&& +\,\gamma \sum _{s^{\prime }\in \mathbb {S}}P^{\pi ^{-i}}(s^{\prime }|s,a^i)V_i^{\pi ^i,\pi ^{-i}}(s^{\prime })\bigg ] \\
&=&\sum _{a^i\in \mathbb {A}^i}\pi ^i(s,a^i)Q_i^{\pi ^i,\pi ^{-i}}(s,a^i) \\
&=&\sum _{a^i\in \mathbb {A}^i_+}\pi ^i(s,a^i)Q_i^{\pi ^i,\pi ^{-i}}(s,a^i).
\end{eqnarray*}$$

Since $\sum _{a^i\in \mathbb {A}^i_+}\pi ^i(s,a^i)=1$, there must exist some $c^{i}\in \mathbb {A}^i_+$ such that $Q_i^{\pi ^i,\pi ^{-i}}(s,c^i) <V_i^{\pi ^i,\pi ^{-i}} (s)$, because otherwise we have $Q_i^{\pi ^i,\pi ^{-i}} (s,a^i)\ge V_i^{\pi ^i,\pi ^{-i}}(s)$ for all $a^i\in \mathbb {A}^i_+$, which, com-bined with $Q_i^{\pi ^i,\pi ^{-i}}(s, d^i) >V_i^{\pi ^i,\pi ^{-i}} (s)$, implies that $\sum _{a^i\in \mathbb {A}^i_+}\pi ^i (s,a^i) Q_i^{\pi ^i,\pi ^{-i}}(s,a^i)>V_i^{\pi ^i,\pi ^{-i}} (s)$, a contradiction to the above equation. With some further calculation, we can have the equation

$$\begin{eqnarray*}
&&(f(\pi ))^i(s,c^i)\\
&& =\frac{\pi ^i(s,c^i)\! +\!\max \left(0,Q_i^{\pi ^i,\pi ^{-i}}(s,c^i)\!-\!V_i^{\pi ^i,\pi ^{-i}}(s)\right)}{1\!+\!\sum _{b^i\in \mathbb {A}^i}\max \left(0,Q_i^{\pi ^i,\pi ^{-i}}(s,b^i)\!-\!V_i^{\pi ^i,\pi ^{-i}}(s)\right)}\\
&& =\frac{\pi ^i(s,c^i)}{1\!+\!\sum _{b^i\in \mathbb {A}^i}\max \left(0,Q_i^{\pi ^i,\pi ^{-i}}(s,b^i)\!-\!V_i^{\pi ^i,\pi ^{-i}}(s)\right)} \\
&& \le \frac{\pi ^i(s,c^i)}{1\!+\!Q_i^{\pi ^i,\pi ^{-i}}(s,d^i)\!-\!V_i^{\pi ^i,\pi ^{-i}}(s)} \\
&& <\pi ^i(s,c^i).
\end{eqnarray*}$$

The above strict inequality follows because $1+Q_i^{\pi ^i,\pi ^{-i}}(s,d^i)-V_i^{\pi ^i,\pi ^{-i}}(s)>1$ as well as π^*i*^(*s, c*^*i*^) > 0.

This contradicts with the assumption that π is a fixed point of *f*. Therefore, it holds for any $a^i\in \mathbb {A}^i$ that $Q_i^{\pi ^i,\pi ^{-i}}(s,a^i)\le V_i^{\pi ^i,\pi ^{-i}}(s)$.□

Combining Claim 5 and Lemma 4 (to be proven next), we find that, for any $\tilde{\pi }^i\in \Delta _{A^i}^S$ and any $s\in \mathbb {S}$, $V_i^{\pi ^i,\pi ^{-i}}(s)\ge V_i^{\tilde{\pi }^i,\pi ^{-i}}(s)$. Thus, π is an MPE by definition. This completes the proof of Theorem 2.□

**Lemma 4.** *For any player i* ∈ [*n*], *given* π^−*i*^, *for any*$\pi ^i\in \Delta _{A^i}^S$, *the following two statements are equivalent*:

1. for all $s\in \mathbb {S}$ and all $a^i\in \mathbb {A}^i$, $V_i^{\pi ^i,\pi ^{-i}}(s)\ge Q_i^{\pi ^i,\pi ^{-i}}(s,a^i)$;
2. for all $s\in \mathbb {S}$ and all $\tilde{\pi }^i\in \Delta _{A^i}^S$, $V_i^{\pi ^i,\pi ^{-i}}(s)\ge V_i^{\tilde{\pi }^i,\pi ^{-i}}(s)$.

*Proof.* Let $\mathbb {V}$ denote the space of value functions $\mathbb {S}\rightarrow \mathcal {R}$, and define the *l*_∞_ norm for any $v\in \mathbb {V}$ as $\Vert v\Vert _\infty =\max _{s\in \mathbb {S}}|v(s)|$.

Pick any player *i* ∈ [*n*] and keep π^−*i*^ fixed. Define the Bellman operator $\Phi ^i:\mathbb {V}\rightarrow \mathbb {V}$ such that, for any $v\in \mathbb {V}$ and any $s\in \mathbb {S}$,

$$\begin{eqnarray*}
\Phi ^i(v)(s)&:=&\max _{a^i\in \mathbb {A}^i}\bigg [r^{i,\pi ^{-i}}(s,a^i)\\
&& +\,\gamma \sum _{s^{\prime }\in \mathbb {S}}P^{\pi ^{-i}}(s^{\prime }|s,a^i)v(s^{\prime })\bigg ].
\end{eqnarray*}$$

Note that, for all $\tilde{\pi }^i\in \Delta _{A^i}^S$, $\Phi ^i(V_i^{\tilde{\pi }^i,\pi ^{-i}})(s) =\max _{a^i\in \mathbb {A}^i} Q_i^{\tilde{\pi }^i,\pi ^{-i}}(s,a^i)$, since $Q_i^{\tilde{\pi }^i,\pi ^{-i}}(s,a^i)= r^{i,\pi ^{-i}}(s,a^i) +\gamma \sum _{s^{\prime }\in \mathbb {S}}P^{\pi ^{-i}}(s^{\prime }|s,a^i)V_i^{\tilde{\pi }^i,\pi ^{-i}}(s^{\prime })$.

We first prove the equivalence between statements 1 and 2, based on Claim 6 below, which will be proved next for completeness.

2⇒1: From statement 2, for all $s\in \mathbb {S}$, $V_i^{\pi ^i,\pi ^{-i}} (s\!)\!=\max _{\tilde{\pi }^i\in \Delta _{A^i}^S}\!V_i^{\tilde{\pi }^i,\pi ^{-i}}(s)\!=v^{i*}(s\!)$, which is the fixed point of Φ^*i*^ by Claim 6 below. That is, for all $s\in \mathbb {S}$, $V_i^{\pi ^i,\pi ^{-i}}(s)=\Phi ^i(V_i^{\pi ^i,\pi ^{-i}})(s)=\max _{a^i\in \mathbb {A}^i} Q_i^{\pi ^i,\pi ^{-i}}(s,a^i)$, by definition of the Bellman operator Φ^*i*^. Statement 1 holds.

1⇒2: If statement 1 holds, we have, for all $s\in \mathbb {S}$, $V_i^{\pi ^i,\pi ^{-i}}(s)\ge \max _{a^i\in \mathbb {A}^i} Q_i^{\pi ^i,\pi ^{-i}}(s,a^i)$. Since $V_i^{\pi ^i,\pi ^{-i}} (s)\le \max _{a^i\in \mathbb {A}^i} Q_i^{\pi ^i,\pi ^{-i}}(s,a^i)$ by Claim 6 below, we get $V_i^{\pi ^i,\pi ^{-i}}(s)=\max _{a^i\in \mathbb {A}^i} Q_i^{\pi ^i,\pi ^{-i}}(s,a^i)$. $\Phi ^i(V_i^{\pi ^i,\pi ^{-i}})(s)=\max _{a^i\in \mathbb {A}^i}Q_i^{\pi ^i,\pi ^{-i}}(s,a^i)=V_i^{\pi ^i,\pi ^{-i}}(s)$, implying that $V_i^{\pi ^i,\pi ^{-i}}$ is a fixed point of Φ^*i*^. By Claim 6, the unique fixed point of Φ^*i*^ is $v^{i*}=V_i^{\pi ^i,\pi ^{-i}}$. Therefore, for all $s\in \mathbb {S}$, $V_i^{\pi ^i,\pi ^{-i}}(s)=\max _{\tilde{\pi }^i\in \Delta _{A^i}^S}V_i^{\tilde{\pi }^i,\pi ^{-i}}(s)$: statement 2 holds.

**Claim 6.** *We have the following important properties*.

- *It holds that* Φ^*i*^*is a* γ*-contraction mapping with respect to the l*_∞_*norm, and has a unique fixed point*.
- *For any* $\tilde{\pi }^i\in \Delta _{A^i}^S$ *and any* $s\in \mathbb {S}$ , $\Phi ^i(V_i^{\tilde{\pi }^i,\pi ^{-i}})(s)\ge V_i^{\tilde{\pi }^i,\pi ^{-i}}(s)$.
- *Let* $v^{i*}\in \mathbb {V}$ *denote the fixed point of* Φ^*i*^; *then v*^i^* *is the optimal value function, i.e. for any*$s\in \mathbb {S}$, $v^{i*}(s)=\max _{\tilde{\pi }^i\in \Delta _{A^i}^S}V_i^{\tilde{\pi }^i,\pi ^{-i}}(s)$.

*Proof of Claim 6*. Define

$$\begin{eqnarray*}
Q_i^{v}(s,a^i)&=&r^{i,\pi ^{-i}}(s,a^i)\\
&& +\, \gamma \sum _{s^{\prime }\in \mathbb {S}}P^{\pi ^{-i}}(s^{\prime }|s,a^i)v(s^{\prime }).
\end{eqnarray*}$$

We have $\Phi ^i(v)(s)=\max _{a^i\in \mathbb {A}^i}Q_i^{v}(s,a^i)$ for all $v\in \mathbb {V}$ and $s\in \mathbb {S}$.

We first prove that Φ^*i*^ is a γ-contraction mapping with respect to the *l*_∞_ norm. For all $v_1,v_2\in \mathbb {V}$, let $\delta =\Vert v_1-v_2\Vert _\infty =\max _{s\in \mathbb {S}}|v_1(s)-v_2(s)|$. We show that ‖Φ^*i*^(*v*_1_) − Φ^*i*^(*v*_2_)‖_∞_ ≤ γδ.

For all $s\in \mathbb {S}$ and all $a^i\in \mathbb {A}^i$, observe that

$$\begin{eqnarray*}
&&Q_i^{v_1}(s,a^i)-Q_i^{v_2}(s,a^i)\\ &&=\gamma \sum _{s^{\prime }\in \mathbb {S}}P^{\pi ^{-i}}(s^{\prime }|s,a^i) (v_1(s^{\prime })-v_2(s^{\prime })),
\end{eqnarray*}$$

so $|Q_i^{v_1}(s,a^i)-Q_i^{v_2}(s,a^i)| \le \gamma \sum _{s^{\prime }\in \mathbb {S}}P^{\pi ^{-i}} (s^{\prime }|s,a^i)\delta =\gamma \delta$.

Without loss of generality, one can suppose that Φ^*i*^(*v*_1_)(*s*) ≥ Φ^*i*^(*v*_2_)(*s*). Taking arbitrary $a_1^i\in \arg \max _{a^i\in \mathbb {A}^i}Q_i^{v_1}(s,a^i)$, we have

$$\begin{eqnarray*}
\Phi ^i(v_2)(s)&=&\max _{a^i\in \mathbb {A}^i}Q_i^{v_2}(s,a^i) \\
&&\ge Q_i^{v_2}\left(s,a_1^i\right) \\
&&\ge Q_i^{v_1}\left(s,a_1^i\right)-\gamma \delta \\
&&=\Phi ^i(v_1)(s)-\gamma \delta ;
\end{eqnarray*}$$

thus, |Φ^*i*^(*v*_1_)(*s*) − Φ^*i*^(*v*_2_)(*s*)| ≤ γδ. By symmetry, the claim holds for the case in which Φ^*i*^(*v*_1_)(*s*) ≤ Φ^*i*^(*v*_2_)(*s*). Therefore, it holds that ‖Φ^*i*^(*v*_1_) − Φ^*i*^(*v*_2_)‖_∞_ ≤ γδ. Thus, Φ^*i*^ is a γ-contraction mapping.

By the Banach fixed point theorem, we know that $\Phi ^i:\mathbb {V}\rightarrow \mathbb {V}$ has a unique fixed point $v^{i*}\in \mathbb {V}$. Moreover, for any $v\in \mathbb {V}$, the point sequence *v*, Φ^*i*^(*v*), Φ^*i*^(Φ^*i*^(*v*)), … converges to *v*^i^*, i.e. for all $s\in \mathbb {S}$, lim_*k* → ∞_(Φ^*i*^)^(*k*)^(*v*)(*s*) = *v*^i^*(*s*), where (Φ^*i*^)^(*k*)^ = Φ^*i*^$\circ$ (Φ^*i*^)^(*k* − 1)^ is defined recursively with (Φ^*i*^)^(1)^ = Φ^*i*^.

Next, for all $\tilde{\pi }^i\in \Delta _{A^i}^S$ and all $s\in \mathbb {S}$, $\Phi ^i(V_i^{\tilde{\pi }^i,\pi ^{-i}})(s)\ge V_i^{\tilde{\pi }^i,\pi ^{-i}}(s)$, since

$$\begin{eqnarray*}
V_i^{\tilde{\pi }^i,\pi ^{-i}}(s)&=&\sum \limits_{a^i\in \mathbb {A}^i}\tilde{\pi }^i(s,a^i) Q_i^{\tilde{\pi }^i,\pi ^{-i}}(s,a^i) \\
&\le& \max _{a^i\in \mathbb {A}^i}Q_i^{\tilde{\pi }^i,\pi ^{-i}}(s,a^i) \\
&=&\Phi ^i(V_i^{\tilde{\pi }^i,\pi ^{-i}})(s)
\end{eqnarray*}$$

by definition.

Finally we prove that, for any $s\in \mathbb {S}$, $v^{i*}(s)=\max _{\tilde{\pi }^i\in \Delta _{A^i}^S}V_i^{\tilde{\pi }^i,\pi ^{-i}}(s)$. For any $\pi ^i\in \Delta _{A^i}^S$, define the operator $\Psi ^i_{\pi ^i}:\mathbb {V}\rightarrow \mathbb {V}$, such that, for any $v\in \mathbb {V}$ and any $s\in \mathbb {S}$,

$$\begin{equation*}
\Psi ^i_{\pi ^i}(v)(s):=\sum _{a^i\in \mathbb {A}^i}\pi ^i(s,a^i)Q_i^v(s,a^i).
\end{equation*}$$

Note that, for any $\pi ^i\in \Delta _{A^i}^S$, $\Psi ^i_{\pi ^i}$ is also a γ-contraction mapping. This is because, for any $v_1,v_2\in \mathbb {V}$ such that ‖*v*_1_ − *v*_2_‖_∞_ = δ, we have shown that, for any $s\in \mathbb {S}$ and any $a^i\in \mathbb {A}^i$, $|Q_i^{v_1}(s,a^i)-Q_i^{v_2}(s,a^i)|\le \gamma \delta$, so

$$\begin{eqnarray*}
&&\left|\Psi ^i_{\pi ^i}(v_1)(s)-\Psi ^i_{\pi ^i}(v_2)(s)\right| \\
&&\quad \le \sum _{a^i\in \mathbb {A}^i}\pi ^i(s,a^i)\left|Q_i^{v_1}(s,a^i)-Q_i^{v_2}(s,a^i)\right| \\
&&\quad \le \gamma \delta ,
\end{eqnarray*}$$

and then $\Vert \Psi ^i_{\pi ^i}(v_1)-\Psi ^i_{\pi ^i}(v_2)\Vert _\infty \le \gamma \delta$.

For any $\pi ^i\in \Delta _{A^i}^S$, we can observe that $V_i^{\pi ^i,\pi ^{-i}}=\Psi ^i_{\pi ^i}(V_i^{\pi ^i,\pi ^{-i}})$ by definition. By the Banach fixed point theorem, we know that $\Psi ^i_{\pi ^i}$ has a unique fixed point in $\mathbb {V}$, so $V_i^{\pi ^i,\pi ^{-i}}$ is exactly the unique fixed point of $\Psi ^i_{\pi ^i}$.

Now we arbitrarily take a policy $\pi _*^i\in \Delta _{A^i}^S$ such that, for all $s\in \mathbb {S}$, $\lbrace a^i\in \mathbb {A}^i:\pi _*^i(s,a^i)>0\rbrace \subseteq \arg \max _{a^i\in \mathbb {A}^i}Q_i^{v^{i*}}(s,a^i)$. It can be seen that, for any$s\in \mathbb {S}$,

$$\begin{eqnarray*}
\Psi ^i_{\pi _*^i}(v^{i*})(s)&=&\max _{a^i\in \mathbb {A}^i}Q_i^{v^{i*}}(s,a^i) \\
&=&\Phi ^i(v^{i*})(s) \\
&=&v^{i*}(s).
\end{eqnarray*}$$

It follows that $\Psi ^i_{\pi _*^i}(v^{i*})=v^{i*}$, so *v*k^i^* is a fixed point of $\Psi ^i_{\pi _*^i}$. Since the unique fixed point of $\Psi ^i_{\pi _*^i}$ is $V_i^{\pi _*^i,\pi ^{-i}}$, we have $V_i^{\pi _*^i,\pi ^{-i}}=v^{i*}$. Thus, for any $s\in \mathbb {S}$, $\max _{\tilde{\pi }^i\in \Delta _{A^i}^S}V_i^{\tilde{\pi }^i,\pi ^{-i}}(s)\ge V_i^{\pi _*^i,\pi ^{-i}}(s)=v^{i*}(s)$.

To show that, for any $s\in \mathbb {S}$, $v^{i*}(s)\ge \max _{\tilde{\pi }^i\in \Delta _{A^i}^S}V_i^{\tilde{\pi }^i,\pi ^{-i}}(s)$, we observe that, given $v_1,v_2\in \mathbb {V}$, if for all $s\in \mathbb {S}, v_1(s)\le v_2(s)$, then, for any $s\in \mathbb {S}$ and any $a^i\in \mathbb {A}^i$, $Q_i^{v_1}(s,a^i)\le Q_i^{v_2}(s,a^i)$. Therefore, $\Phi ^i(v_1)(s)=\max _{a^i\in \mathbb {A}^i}Q_i^{v_1}(s,a^i)\le \max _{a^i\in \mathbb {A}^i}Q_i^{v_2}(s,a^i) =\Phi ^i(v_2)(s)$. As we have, for all $\tilde{\pi }^i\in \Delta _{A^i}^S$ and all $s\in \mathbb {S}$, $\Phi ^i(V_i^{\tilde{\pi }^i,\pi ^{-i}})(s)\ge V_i^{\tilde{\pi }^i,\pi ^{-i}}(s)$, we have, for any $k\in \mathcal {N}$ and any $s\in \mathbb {S}$, $(\Phi ^i)^{(k+1)}(V_i^{\tilde{\pi }^i,\pi ^{-i}})(s)\ge (\Phi ^i)^{(k)}(V_i^{\tilde{\pi }^i,\pi ^{-i}})(s)$ by induction. It follows that $V_i^{\tilde{\pi }^i,\pi ^{-i}}(s)\le (\Phi ^i)^{(k+1)}(V_i^{\tilde{\pi }^i,\pi ^{-i}})(s)$. Let *k* → ∞; then we get $V_i^{\tilde{\pi }^i,\pi ^{-i}}(s)\le \lim _{k\rightarrow \infty }(\Phi ^i)^{(k)}(V_i^{\tilde{\pi }^i,\pi ^{-i}})(s)=v^{i*}(s)$. Thus, $v^{i*}(s)\ge \max _{\tilde{\pi }^i\in \Delta _{A^i}^S}V_i^{\tilde{\pi }^i,\pi ^{-i}}(s)$.

The claim that, for all $s\in \mathbb {S}$, $v^{i*}(s)=\max _{\tilde{\pi }^i\in \Delta _{A^i}^S}V_i^{\tilde{\pi }^i,\pi ^{-i}}(s)$ follows.

## THE APPROXIMATION GUARANTEE

Theorem 2 states that π is a fixed point of *f* if and only if π is an MPE for the stochastic game. Now we prove that *f* has some good approximation properties: if we find an ε-approximate fixed point π of *f* then it is also a $\mathrm{poly}(|\mathcal {SG}|)\sqrt{\epsilon }$-approximate MPE for the stochastic game (combining the following Lemma 5 and Lemma 6). This implies the **PPAD**-membership of Approximate MPE.

**Lemma 5.** *Let* ε > 0 *and* π *be a strategy profile. If* ‖*f*(π) − π‖_∞_ ≤ ε *then, for each player i* ∈ [*n*] *and each state*$s\in \mathbb {S}$, *we have*

$$\begin{eqnarray*}
&&\max _{a^i\in \mathbb {A}^i}\left(Q_i^{\pi ^i,\pi ^{-i}}(s,a^i)-V_i^{\pi ^i,\pi ^{-i}}(s)\right) \\
&&\quad \le A_{\max }\left(r_{\max }\sqrt{\epsilon ^{\prime }}+\frac{\sqrt{\epsilon ^{\prime }}}{1-\gamma }+\epsilon ^{\prime }\right),
\end{eqnarray*}$$

where

$$\begin{equation*}
\epsilon ^{\prime }=\epsilon \bigg (1+\frac{A_{\max }r_{\max }}{1-\gamma }\bigg ).
\end{equation*}$$

*Proof.* Pick any player *i* ∈ [*n*] and any state $s\in \mathbb {S}$. For simplicity, for any $a^i\in \mathbb {A}^i$, define $Q(a^i)=Q_i^{\pi ^i,\pi ^{-i}}(s,a^i)$ and $M(a^i)=\max (0,Q(a^i)-V_i^{\pi ^i,\pi ^{-i}}(s))$.

First we give an upper bound on *M*(*a*^*i*^). For any $a^i\in \mathbb {A}^i$, it can be easily seen that

$$\begin{equation*}
M(a^i)\le Q(a^i)=Q_i^{\pi ^i,\pi ^{-i}}(s,a^i)\le \frac{r_{\max }}{1-\gamma }.
\end{equation*}$$

By the condition ‖*f*(π) − π‖_∞_ ≤ ε , for any $a^i\in \mathbb {A}^i$, we have

$$\begin{eqnarray*}
&&\pi ^i(s,a^i)-\frac{\pi ^i(s,a^i)+M(a^i)}{1+\sum _{b^i\in \mathbb {A}^i}M(b^i)}\le \epsilon \\
\Rightarrow \quad && \pi ^i(s,a^i)\sum _{b^i\in \mathbb {A}^i}M(b^i)-M(a^i)\\
&& \le \bigg (1+\sum _{b^i\in \mathbb {A}^i}M(b^i)\bigg )\epsilon \\
\Rightarrow \quad && \pi ^i(s,a^i)\sum _{b^i\in \mathbb {A}^i}M(b^i)\\
&& \le M(a^i)+ \bigg (1+\frac{A_{\max }r_{\max }}{1-\gamma }\bigg )\epsilon .
\end{eqnarray*}$$

Set ε′ = (1 + *A*_max _*r*_max _/(1 − γ))ε; then we have the crucial inequality

(1) \begin{equation*} \pi ^i(s,a^i)\sum _{b^i\in \mathbb {A}^i}M(b^i)\le M(a^i)+\epsilon ^{\prime }. \end{equation*}

Let $\mathbb {A}_{-}^i$ denote $\lbrace a^i\in \mathbb {A}^i:M(a^i)=0\rbrace$ or, equivalently, $\lbrace a^i\in \mathbb {A}^i:Q(a^i)-V_i^{\pi ^i,\pi ^{-i}}(a^i)\le 0\rbrace$. Let $t=\sum _{a^i\in \mathbb {A}_{-}^i}\pi ^i(s,a^i)$.

*Case 1: $t\ge \sqrt{\epsilon ^{\prime }}/r_{\max }$.* By inequality (1) we have

$$\begin{eqnarray*}
&&\sum _{a^i\in \mathbb {A}_{-}^i}\pi ^i(s,a^i)\sum _{b^i\in \mathbb {A}^i}M(b^i)\\
&&\le \sum _{a^i\in \mathbb {A}_{-}^i} (M(a^i)+\epsilon ^{\prime }) \\
\Rightarrow \quad && t\sum _{b^i\in \mathbb {A}^i}M(b^i)\le \sum _{a^i\in \mathbb {A}_{-}^i}\epsilon ^{\prime }\\
\Rightarrow \quad && \sum _{b^i\in \mathbb {A}^i}M(b^i)\\
&& \le A_{\max }\epsilon ^{\prime }/t=A_{\max }r_{\max }\sqrt{\epsilon ^{\prime }}.
\end{eqnarray*}$$

*Case 2: $t<\sqrt{\epsilon ^{\prime }}/r_{\max }$.* By inequality (1) we have

(2) \begin{eqnarray*} &&\pi ^i(s,a^i)\sum _{b^i\in \mathbb {A}^i}M(b^i)\le M(a^i)+\,\epsilon ^{\prime }\nonumber\\ && \quad \text{for all } a^i\in \mathbb {A}^{i}\nonumber \\ \Rightarrow && \sum _{a^i\in \mathbb {A}^{i}}(\pi ^i(s,a^i))^2\sum _{b^i\in \mathbb {A}^i}M(b^i)\nonumber \\ &&\quad \le \sum _{a^i\in \mathbb {A}^{i}}\pi ^i(s,a^i)M(a^i)+\epsilon ^{\prime }\nonumber \\ \Rightarrow && \sum _{a^i\in \mathbb {A}^{i}}(\pi ^i(s,a^i))^2\sum _{b^i\in \mathbb {A}^i}M(b^i)\nonumber \\ &&\quad \le \sum _{a^i\in \mathbb {A}^i\setminus \mathbb {A}_{-}^i}\pi ^i(s,a^i)M(a^i)+\epsilon ^{\prime }. \end{eqnarray*}

As $V_i^{\pi ^i,\pi ^{-i}}(s)=\sum _{a^i\in \mathbb {A}^i}\pi ^i(s,a^i)Q(a^i)$ and, for all $a^i\in \mathbb {A}^i\setminus \mathbb {A}_{-}^i$, $M(a^i)=Q(a^i)-V_i^{\pi ^i,\pi ^{-i}}(s)$,

$$\begin{eqnarray*}
0&=&\sum _{a^i\in \mathbb {A}^{i}}\pi ^i(s,a^i)(Q(a^i)-V_i^{\pi ^i,\pi ^{-i}}(s))\\
&=&\sum _{a^i\in \mathbb {A}^{i}\setminus \mathbb {A}_{-}^i}\pi ^i(s,a^i)M(a^i)\\
&& +\sum _{a^i\in \mathbb {A}_{-}^i}\pi ^i(s,a^i)(Q(a^i)-V_i^{\pi ^i,\pi ^{-i}}(s)).
\end{eqnarray*}$$

Therefore,

$$\begin{eqnarray*}
& &\sum _{a^i\in \mathbb {A}^{i}\setminus \mathbb {A}_{-}^i}\pi ^i(s,a^i)M(a^i)\\
&&\quad =\sum _{a^i\in \mathbb {A}_{-}^i}\pi ^i(s,a^i)(V_i^{\pi ^i,\pi ^{-i}}(s)-Q(a^i))\\
&&\quad \le \frac{r_{\max }}{1-\gamma }t\\
&&\quad <\frac{\sqrt{\epsilon ^{\prime }}}{1-\gamma }.
\end{eqnarray*}$$

Moreover, observe that $\sum _{a^i\in \mathbb {A}^i}(\pi ^i(s,a^i))^2\ge 1/{A_{\max }}$. Substituting these into inequality (2), we get

$$\begin{equation*}
\frac{1}{A_{\max }}\sum _{b^i\in \mathbb {A}^i}M(b^i)\le \frac{\sqrt{\epsilon ^{\prime }}}{1-\gamma }+\epsilon ^{\prime }.
\end{equation*}$$

It follows that $\sum _{b^i\in \mathbb {A}^i}M(b^i)\le A_{\max }({\sqrt{\epsilon ^{\prime }}}/ {(1-\gamma )}+\epsilon ^{\prime })$.

In conclusion, combining the two cases, we get

$$\begin{equation*}
\sum _{b^i\in \mathbb {A}^i}M(b^i)\le A_{\max }\bigg (r_{\max }\sqrt{\epsilon ^{\prime }}+\frac{\sqrt{\epsilon ^{\prime }}}{1-\gamma }+\epsilon ^{\prime }\bigg ).
\end{equation*}$$

Thus, for each $a^i\in \mathbb {A}^i$, we have

$$\begin{eqnarray*}
&&Q_i^{\pi ^i,\pi ^{-i}}(s,a^i)-V_i^{\pi ^i,\pi ^{-i}}(s) \le M(a^i) \\
&&\quad \le \sum _{b^i\in \mathbb {A}^i}M(b^i) \\
&&\quad \le A_{\max }\bigg (r_{\max }\sqrt{\epsilon ^{\prime }}+\frac{\sqrt{\epsilon ^{\prime }}}{1-\gamma }+\epsilon ^{\prime }\bigg ),
\end{eqnarray*}$$

which completes the proof.□

**Lemma 6.** *Let* ε > 0 *and* π *be a strategy profile. If, for each player i* ∈ [*n*] *and each state*$s\in \mathbb {S}$, $\max _{a^i\in \mathbb {A}^i}Q_i^{\pi ^i,\pi ^{-i}}(s,a^i)-V_i^{\pi ^i,\pi ^{-i}}(s)\le \epsilon$*then* π *is an* ε/(1 − γ)*-approximate MPE*.

*Proof.* Recall the mapping $\Phi ^i:\mathbb {V}\rightarrow \mathbb {V}$, defined as the Bellman operator, from the proof of Lemma 4. Let $v^{i*}\in \mathbb {V}$ be the unique fixed point of Φ^*i*^ and recall that, for all $s\in \mathbb {S}$, $v^{i*}(s)=\max _{\tilde{\pi }^i\in \Delta _{A^i}^S}V_i^{\tilde{\pi }^i,\pi ^{-i}}(s)$.

Pick any player *i* ∈ [*n*]; by assumption, for each state $s\in \mathbb {S}$, we have $\max _{a^i\in \mathbb {A}^i}Q_i^{\pi ^i,\pi ^{-i}}(s,a^i)-V_i^{\pi ^i,\pi ^{-i}}(s)\le \epsilon$. On the other hand, $V_i^{\pi ^i,\pi ^{-i}}(s)\le \max _{a^i\in \mathbb {A}^i}Q_i^{\pi ^i,\pi ^{-i}}(s,a^i)$, so we have $|V_i^{\pi ^i,\pi ^{-i}}(s)-\max _{a^i\in \mathbb {A}^i}Q_i^{\pi ^i,\pi ^{-i}}(s,a^i)|\le \epsilon$, i.e. $\Vert V_i^{\pi ^i,\pi ^{-i}}-\Phi ^i(V_i^{\pi ^i,\pi ^{-i}})\Vert _\infty \le \epsilon$.

Since Φ^*i*^ is a γ-contraction mapping,

$$\begin{eqnarray*}
&&\Vert v^{i*}-\Phi ^i(V_i^{\pi ^i,\pi ^{-i}})\Vert _\infty\\
&&\quad =\Vert \Phi ^i(v^{i*})-\Phi ^i(V_i^{\pi ^i,\pi ^{-i}})\Vert _\infty \\
&&\quad\le \gamma \Vert v^{i*}-V_i^{\pi ^i,\pi ^{-i}}\Vert _\infty .
\end{eqnarray*}$$

In addition, by the triangle inequality we have

$$\begin{eqnarray*}
&&\Vert v^{i*}-\Phi ^i(V_i^{\pi ^i,\pi ^{-i}})\Vert _\infty\\
&&\qquad +\,\Vert V_i^{\pi ^i,\pi ^{-i}}-\Phi ^i(V_i^{\pi ^i,\pi ^{-i}})\Vert _\infty \\
&&\quad \ge \Vert v^{i*}-V_i^{\pi ^i,\pi ^{-i}}\Vert _\infty ,
\end{eqnarray*}$$

so it follows that

$$\begin{eqnarray*}
&&\Vert V_i^{\pi ^i,\pi ^{-i}}-\Phi ^i(V_i^{\pi ^i,\pi ^{-i}})\Vert _\infty\\
&&\quad \ge (1-\gamma )\Vert v^{i*}-V_i^{\pi ^i,\pi ^{-i}}\Vert _\infty .
\end{eqnarray*}$$

Thus, we have

$$\begin{eqnarray*}
\Vert v^{i*}-V_i^{\pi ^i,\pi ^{-i}}\Vert _\infty &\le &\frac{1}{1-\gamma }\Vert V_i^{\pi ^i,\pi ^{-i}}\\
&& -\, \Phi ^i(V_i^{\pi ^i,\pi ^{-i}})\Vert _\infty \\
&\le & \frac{\epsilon }{1-\gamma } .
\end{eqnarray*}$$

It follows that, for any $s\in \mathbb {S}$ and any $\tilde{\pi }^i\in \Delta _{A^i}^S$,

$$\begin{eqnarray*}
&&V_i^{\tilde{\pi }^i,\pi ^{-i}}(s)-V_i^{\pi ^i,\pi ^{-i}}(s)\\
&&\quad \le v^{i*}(s)-V_i^{\pi ^i,\pi ^{-i}}(s)\\
&&\quad \le \Vert v^{i*}-V_i^{\pi ^i,\pi ^{-i}}\Vert _\infty \\
&&\quad \le \frac{\epsilon }{1-\gamma }.
\end{eqnarray*}$$

By definition, it follows that π is an ε/(1 − γ)-approximate MPE.□

To conclude, Lemma 2 is proven by combining Lemma 5 and Lemma 6, which completes the proof of Lemma 1.

## CONCLUSION

Solving an MPE in general-sum SGs has long expected to be at least **PPAD**-hard. In this paper, we prove that computing an MPE in a finite-state infinite horizon discounted SG is **PPAD**-complete. Our completeness result also immediately implies the **PPAD**-completeness of computing an MPE in action-free SGs and single-controller SGs. We hope that our results can encourage MARL researchers to study solving an MPE in general-sum SGs, proposing a sample-efficient MARL solution, which leads to more prosperous algorithmic developments than those currently on zero-sum SGs.

## APPENDIX

### Proof of Claim 1

We have

$$\begin{eqnarray*}
&&|(x+y)(1+z^{\prime })-(x^{\prime }+y^{\prime })(1+z)|\\
&&\quad \le |(x+y)(1+z^{\prime })-(x^{\prime }+y^{\prime })(1+z^{\prime })|\\
&&\quad \quad +|(x^{\prime }+y^{\prime })(1+z^{\prime })-(x^{\prime }+y^{\prime })(1+z)|\\
&&\quad = (1+z^{\prime })|(x+y)-(x^{\prime }+y^{\prime })|\\
&&\quad +(x^{\prime }+y^{\prime })|z^{\prime }-z|\\
&&\quad \le (1+z^{\prime })(|x-x^{\prime }+y-y^{\prime }|+|z-z^{\prime }|)\\
&&\quad \le (1+z^{\prime })(|x-x^{\prime }|+|y-y^{\prime }|+|z-z^{\prime }|)\\
&& \le (1+z)(1+z^{\prime })(|x-x^{\prime }|+|y-y^{\prime }|+|z-z^{\prime }|).
\end{eqnarray*}$$

The first and third inequalities follow by the triangle inequality, the second inequality holds because *x*′ + *y*′ ≤ 1 + *z*′ and the last inequality follows because 1 + *z* ≥ 1. It immediately follows that

$$\begin{eqnarray*}
&&\bigg |\frac{x+y}{1+z}-\frac{x^{\prime }+y^{\prime }}{1+z^{\prime }}\bigg |\\
&&\quad =\frac{|(x+y)(1+z^{\prime })-(x^{\prime }+y^{\prime })(1+z)|}{(1+z)(1+z^{\prime })}\\
&&\quad \le |x-x^{\prime }|+|y-y^{\prime }|+|z-z^{\prime }|.
\end{eqnarray*}$$

### Proof of Claim 2

We have

$$\begin{eqnarray*}
&&|r^{i,\pi _1^{-i}}(s,b^i)-r^{i,\pi _2^{-i}}(s,b^i)|\\
&& =\bigg |\sum _{b^{-i}\in \mathbb {A}^{-i}}r^i(s,b^i,b^{-i})\pi _1^{-i}(s,b^{-i})\\
&&\quad -\,\sum _{b^{-i}\in \mathbb {A}^{-i}}r^i(s,b^i,b^{-i})\pi _2^{-i}(s,b^{-i})\bigg |\\
&& = \bigg |\sum _{b^{-i}\in \mathbb {A}^{-i}}r^i(s,b^i,b^{-i})(\pi _1^{-i}(s,b^{-i})\\
&& \qquad -\,\pi _2^{-i}(s,b^{-i}))\bigg |\\
&& =\bigg |\sum _{b^{-i}\in \mathbb {A}^{-i}}r^i(s,b^i,b^{-i})\\
&&\qquad \times\bigg (\prod _{j\ne i}\pi _1^{j}(s,b^j)-\prod _{j\ne i}\pi _2^{-i}(s,b^j)\bigg )\bigg |\\
&& \le \sum _{b^{-i}\in \mathbb {A}^{-i}}r^i(s,b^i,b^{-i})\bigg |\prod _{j\ne i}\pi _1^{j}(s,b^j)\\
&&\qquad -\prod _{j\ne i}\pi _2^{j}(s,b^j)\bigg |\\
&& \le r_{\max }\sum _{b^{-i}\in \mathbb {A}^{-i}} \bigg |\prod _{j\ne i}\pi _1^{j}(s,b^j)-\prod _{j\ne i}\pi _2^{j}(s,b^j)\bigg |\\
&& \le (n-1)A_{\max }r_{\max }\delta ,
\end{eqnarray*}$$

where the last inequality follows from the next claim.

### Proof of Claim 3

We have

$$\begin{eqnarray*}
&&\sum _{b^{-1}\in \mathbb {A}^{-1}} \bigg |\prod _{j=2}^n\pi _1^{j}(s,b^j)-\prod _{j=2}^n\pi _2^{j}(s,b^j)\bigg | \\
&&\quad =\sum _{b^{-1}\in \mathbb {A}^{-1}}\bigg |\sum _{k=2}^n\bigg (\prod _{l=2}^{k-1}\pi _1^l(s,b^l)\bigg ) \\
&&\qquad \times (\pi _1^k(s,b^k)-\pi _2^k(s,b^k)) \prod _{l=k+1}^{n}\pi _2^l(s,b^l)\bigg | \\
&&\quad \le \sum _{k=2}^n\sum _{b^{-1}\in \mathbb {A}^{-1}}\bigg (\prod _{l=2}^{k-1}\pi _1^l(s,b^l)\bigg ) \\
&&\qquad \times |\pi _1^k(s,b^k)-\pi _2^k(s,b^k)| \prod _{l=k+1}^{n}\pi _2^l(s,b^l) \\
&&\quad =\sum _{k=2}^n\sum _{b^k\in \mathbb {A}^k}|\pi _1^k(s,b^k)-\pi _2^k(s,b^k)| \\
&& \quad \le (n-1)A_{\max }\delta .
\end{eqnarray*}$$

### Proof of Claim 4

We have

$$\begin{eqnarray*}
&&\sum _{b^{-1}\in \mathbb {A}^{-1}} |P^{\pi _1^{-i}}(s^{\prime }|s,b^i)-P^{\pi _2^{-i}}(s^{\prime }|s,b^i)| \\
&& \le (n-1)A_{\max }\delta |P^{\pi _1^{-i}}(s^{\prime }|s,b^i)-P^{\pi _2^{-i}}(s^{\prime }|s,b^i)| \\
&&\quad =\bigg |\sum _{b^{-i}\in \mathbb {A}^{-i}}P(s^{\prime }|s,b^i,b^{-i})\pi _1^{-i}(s,b^{-i}) \\
&&\qquad -\,\sum _{b^{-i}\in \mathbb {A}^{-i}}P(s^{\prime }|s,b^i,b^{-i})\pi _2^{-i}(s,b^{-i})\bigg | \\
&&\quad =\bigg |\sum _{b^{-i}\in \mathbb {A}^{-i}}P(s^{\prime }|s,b^i,b^{-i})(\pi _1^{-i}(s,b^{-i})\\
&&\qquad -\pi _2^{-i} (s,b^{-i}))\bigg | \\
&&\quad \le \sum _{b^{-i}\in \mathbb {A}^{-i}}P(s^{\prime }|s,b^i,b^{-i})\bigg |\prod _{j\ne i}\pi _1^{j}(s,b^j)\\
&&\qquad-\prod _{j\ne i}\pi _2^{j}(s,b^j)\bigg | \\
&&\quad \le \sum _{b^{-i}\in \mathbb {A}^{-i}}\bigg |\prod _{j\ne i}\pi _1^{j}(s,b^j)-\prod _{j\ne i}\pi _2^{j}(s,b^j)\bigg | \\
&&\quad \le (n-1)A_{\max }\delta .
\end{eqnarray*}$$

### Lemma 7 and its proof

**Lemma 7.** *For every* $\pi _1,\pi _2\in \prod _{i=1}^n\Delta _{A^i}^S$ *such that* ‖π_1_ − π_2_‖_∞_ ≤ δ, *we have*

$$\begin{eqnarray*}
&&|(I-\gamma P^{\pi _1})^{-1}(s^{\prime }|s)-(I-\gamma P^{\pi _2})^{-1}(s^{\prime }|s)|\\
&& \le \frac{nSA_{\max }\delta }{(1-\gamma )^2}
\end{eqnarray*}$$

*for any*$s,s^{\prime }\in \mathbb {S}$.

*Proof.* We first give an upper bound on $|P^{\pi _1}(s^{\prime }|s)-P^{\pi _2}(s^{\prime }|s)|$ for any $s,s^{\prime }\in \mathbb {S}$:

$$\begin{eqnarray*}
&& |P^{\pi _1}(s^{\prime }|s)-P^{\pi _2}(s^{\prime }|s)| \\
&&\quad = \bigg |\sum _{a\in \mathbb {A}}P(s^{\prime }|s,a)\prod _{i\in [n]}\pi _1^i(s,a^i) \\
&&-\sum _{a\in \mathbb {A}}P(s^{\prime }|s,a)\prod _{i\in [n]}\pi _2^i(s,a^i)\bigg | \\
&&\!\!\!\!\! \le \sum _{a\in \mathbb {A}}P(s^{\prime }|s,a)\bigg |\prod _{i\in [n]}\pi _1^i(s,a^i)-\prod _{i\in [n]}\pi _2^i(s,a^i)\bigg | \\
&&\quad \le nA_{\max }\delta .
\end{eqnarray*}$$

Now we view *P*^π^ as an *S* × *S* matrix. For any two *S* × *S* matrices *M*^1^, *M*^2^, we use ‖*M*^1^ − *M*^2^‖_max_ to denote max _*i, j*_|*M*^1^(*i, j*) − *M*^2^(*i, j*)|, i.e. the max norm. Then we have $\Vert P^{\pi _1}-P^{\pi _2}\Vert _{\max }\le nA_{\max }\delta$. Let $Q^1=(I-\gamma P^{\pi _1})^{-1}$ and $Q^2=(I-\gamma P^{\pi _2})^{-1}$. (Note that the inverse of (*I* − γ*P*^π^) must exist because γ < 1.) By definition, we have $Q^1=I+\gamma P^{\pi _1}Q^1$ and $Q^2=I+\gamma P^{\pi _2}Q^2$. Then

$$\begin{eqnarray*}
&&\Vert Q^1-Q^2\Vert _{\max } \\
&&\quad = \gamma \Vert P^{\pi _1}Q^1-P^{\pi _2}Q^2\Vert _{\max } \\
&&\quad = \gamma \max _{i,j}\bigg |\sum _{k}P^{\pi _1}(i,k)Q^1(k,j) \\
&& -\sum _{k}P^{\pi _2}(i,k)Q^2(k,j)\bigg | \\
&&\quad \le \gamma \max _{i,j}\sum _{k}\bigg |P^{\pi _1}(i,k)Q^1(k,j) \\
&&-P^{\pi _2}(i,k)Q^2(k,j)\bigg | \\
&& \le \gamma \max _{i,j}\bigg (\sum _{k}P^{\pi _1}(i,k)|Q^1(k,j)-Q^2(k,j)| \\
&&+\sum _{k}|Q^2(k,j)||P^{\pi _1}(i,k)-P^{\pi _2}(i,k)|\bigg ) \\
&&\quad \le \gamma \max _{i,j}\bigg (\max _{k}|Q^1(k,j)-Q^2(k,j)| \\
&&+\sum _{k}\frac{nA_{\max }\delta }{1-\gamma }\bigg ) \\
&&\quad = \gamma \bigg (\Vert Q^1-Q^2\Vert _{\max }+\frac{nSA_{\max }\delta }{1-\gamma }\bigg ),
\end{eqnarray*}$$

where the sixth line follows the following facts:

1. $\sum _k P^{\pi _1}(i,k)=1$ ;
2. |*Q*^1^(*k, j*) − *Q*^2^(*k, j*)| ≤ max _*k*_|*Q*^1^(*k, j*) − *Q*^2^(*k, j*)|;
3. $|P^{\pi _1}(i,k)-P^{\pi _2}(i,k)|\le nA_{\max }\delta$ ;
4. $|Q^2(k,j)|\le \Vert Q^2\Vert _1\le {1}/{(1-\gamma \Vert P^{\pi _2}\Vert _1)}\le {1}/{(1-\gamma )}$ .

Note that $Q^2=I+\gamma P^{\pi _2}Q^2$. Since the 1-norm is submultiplicative, we have

$$\begin{eqnarray*}
\Vert Q^2\Vert _1&\le &1+\gamma \Vert P^{\pi _2}Q^2\Vert _1 \\
&\le& 1+\gamma \Vert P^{\pi _2}\Vert _1\Vert Q^2\Vert _1 \\
&\le& 1+\gamma \Vert Q^2\Vert _1,
\end{eqnarray*}$$

which leads to the fourth fact. So we have

$$\begin{equation*}
|Q^1-Q^2|_{\max }\le \frac{nSA_{\max }\delta }{(1-\gamma )^2}.
\end{equation*}$$

This completes the proof.
