# Corrigendum to On the complexity of computing Markov perfect equilibrium in general-sum stochastic games

**DOI:** 10.1093/nsr/nwad024

**Published:** 2023-02-01

**Authors:** Xiaotie Deng, Ningyuan Li, David Mguni, Jun Wang, Yaodong Yang

**Affiliations:** Center on Frontiers of Computing Studies, School of Computer Science, Peking University, Beijing 100091, China; Center for Multi-Agent Research, Institute for AI, Peking University, Beijing 100091, China; Center on Frontiers of Computing Studies, School of Computer Science, Peking University, Beijing 100091, China; Huawei UK, London WC1E 6BT, UK; Computer Science, University College London, London WC1E 6BT, UK; Center for Multi-Agent Research, Institute for AI, Peking University, Beijing 100091, China

Xiaotie Deng, Ningyuan Li and Yaodong Yang are corresponding authors of ‘On the complexity of computing Markov perfect equilibrium in general-sum stochastic games’ (*National Science Review*, Volume 10, Issue 1, 2023, https://doi.org/10.1093/nsr/nwac256). In the original published version of this manuscript, only Xiaotie Deng is listed as the corresponding author. The published manuscript has now been corrected to also include Ningyuan Li and Yaodong Yang as corresponding authors.

